# Dental Abnormalities in Pituitary Dwarfism: A Case Report and Review of the Literature

**DOI:** 10.1155/2017/5849173

**Published:** 2017-03-28

**Authors:** Franco Ferrante, Sergio Blasi, Rolando Crippa, Francesca Angiero

**Affiliations:** Department of Surgical Medical Sciences and Integrated Diagnostic, University of Genoa, Genoa, Italy

## Abstract

Hypopituitarism is a disorder caused by a reduced level of trophic hormones that may be consequent on different destructive processes. The clinical manifestations depend on the type of hormone involved. A deficiency of growth hormone (GH) in children causes the lack of growth known as pituitary dwarfism. The case is reported of a patient with pituitary dwarfism, multiple dental anomalies, functional prosthetic problems, and a revision of the literature. She was subjected to prosthetic rehabilitation without surgical intervention, using zirconium substructures, thus eliminating the potential complications that may require trauma surgery. The therapeutic approach adopted led to excellent results and restored an aesthetic smile.

## 1. Introduction

Pituitary dwarfism is a disease resulting when the body fails to use the pituitary growth hormone GH. Adenohypophysary hypofunction occurs when there is parenchymal loss of 75% or more. The causes may be congenital (rare) or acquired. The most frequent causes are nonsecreting adenomas, ischemic necrosis of the pituitary gland, and surgical removal or irradiation of the pituitary adenohypophysis. Hormone deficiency may be the consequence of no or decreased production, or inadequate absorption by the tissues responsible [1]. The disease may thus be expressed either in childhood or in adulthood, the specific clinical picture depending on the period of onset [2, 3]. In children, the failure to use hormones leads to a situation characterized by small size underdeveloped forehead or retrognathism, small nose, childish face, and thin skin [4].

The skeletal age is below the stature age but above the chronological age. The hormone stimulates protein synthesis and thus tissue growth, including that of bone. However, since deficiency of growth hormone is not a thanatophoric condition, the patient may reach adulthood, although he/she will be marked by the characteristics acquired in childhood, such as height below 120 cm, which is the maximum attainable. The poor development of the forehead may lead to the maxilla and mandible being abnormally small, so that the physiological changes associated with tooth eruption may lead to tooth malformation [5–7].

In pituitary dwarfism there may be various anomalies of the dental apparatus, from the morphological profile and in terms of development. The clinical picture presents aspects of hypodontia, delayed tooth eruption, abnormalities of tooth shape and size, and double or impacted teeth.

A case of pituitary dwarfism with dental abnormalities is presented, outlining the clinical therapeutic profile and reviewing the literature.

## 2. Case Presentation

**Figure 1:**
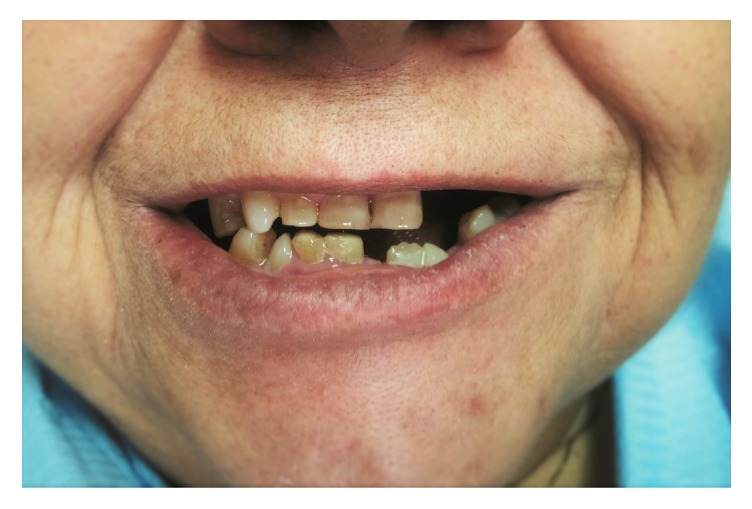
Initial clinical situation.

**Figure 2:**
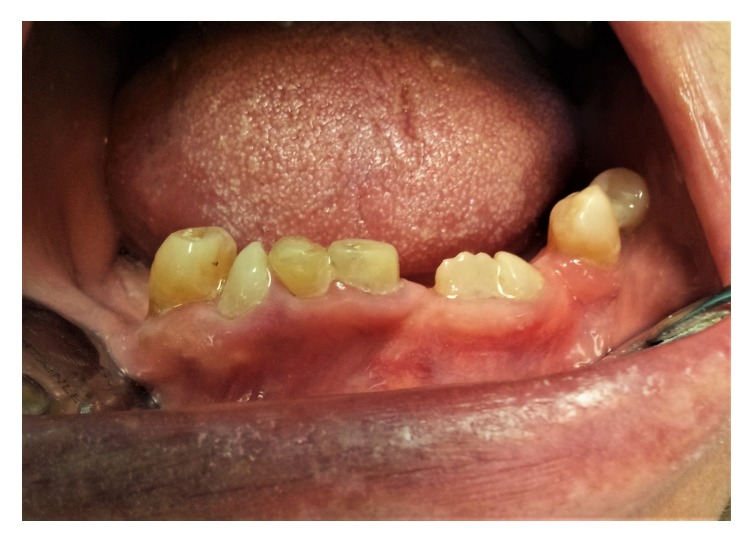
The initial situation at greater magnification.

**Figure 3:**
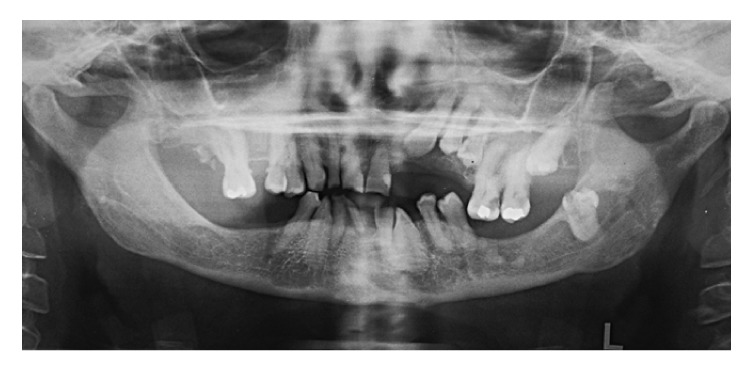
Orthopantomography revealed multiple dental anomalies: hypodontia, radicular fusion of 3.2–3.3, and impacted permanent teeth.

A woman of 50 with very short stature presented to the Department of Prosthetic Dentistry, University of Genoa (Italy) with occlusion problems. She was affected by pituitary dwarfism. Hypodontia together with multiple abnormalities, including tooth shape, small size, and double teeth, was noted during physical examination of the oral cavity (Figures 1 and 2). Orthopantomography was indicated and revealed five impacted teeth and, in the mandibular arch, fused roots of left lateral incisor and left canine (Figure 3). Fusion was present at the radicular level, whereas the crowns were completely separate and independent. As a consequence of the pathological condition of the oral cavity, especially of the teeth, a therapeutic approach was suggested to the patient, with fixed rehabilitation; this proposal was accepted. The patient rejected any form of removable partial denture, after having worn one some years previously for a few months, and preferred a fixed-type rehabilitation. The proposal comprised a five-tooth fixed ceramic prosthesis supported on a six-fixture zirconium substructure for the mandibular arch. The teeth involved were left lateral incisor, left canine, right central incisor, right lateral incisor, and right canine.

**Figure 4:**
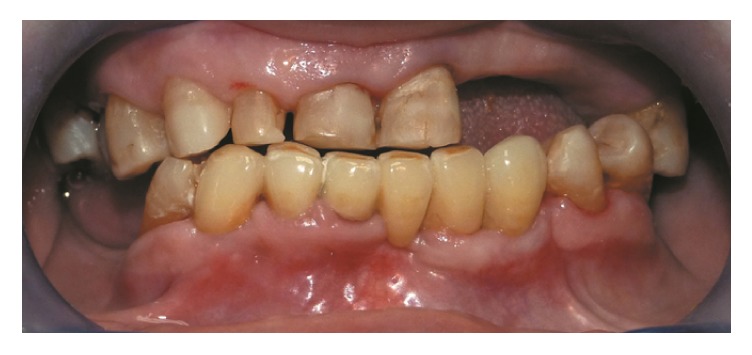
The final phase of prosthetic rehabilitation.

Stumping with cutters was next done, in order to chamfer the teeth in question to 45°, while respecting the parallelism of their interproximal sides; as little dental tissue as possible was removed. After making impressions of the teeth with precision material, a temporary prosthesis was prepared; once the substructure in zirconium was put in place, the occlusion in the anterior region was checked. Having established the validity of the prosthesis, its definitive realization in zirconium-ceramic was accomplished. The result gave the patient a marked and decisive improvement of her smile (Figure 4).

## 3. Discussion

Growth hormones are produced by the anterior lobe of the pituitary gland, which also expresses and secretes five additional hormones (prolactin, thyroid stimulating hormone, follicle stimulating hormone, luteinizing hormone, and adrenocorticotropic hormone). Proper development of the pituitary gland assures the regulation of critical processes, including metabolic control, puberty and reproduction, stress response, and lactation. Disordered expression or function of some genes has been implicated in the etiology of combined pituitary hormone deficiency; these genes act at different stages of pituitary development, resulting in unique patterns of hormonal deficiency reflecting their differential expression during organogenesis. The marked variability in the clinical presentation of patients illustrates the influence of genetic background and environmental factors. However, in the majority of pituitary disorder cases, the etiology of this heterogeneous disease remains unexplained, which suggests additional genes may be involved. Identification of these might also enhance understanding of pituitary development, maintenance, and function.

A literature search (searching in PubMed for “oral cavity hypopituitarism”) produced 57 cases with the presence of numerous anomalies (in particular 13 abnormalities in pituitary dwarfism). Their frequencies, updated to the present time, are presented in Table 1.

Bretéché et al. report that a prevalent anomaly is the presence of a single maxillary median incisor [8]. Although this type of agenesis might be an isolated phenomenon, it might also (as with certain median structure anomalies) be associated with pituitary dwarfism, since this condition occurred in almost 60% of cases of dwarfism [5–8].

Kosowicz and Rzymski identified many other abnormal situations in the oral cavity that recur relatively frequently in pituitary dwarfism [9]. Cobourne and Sharpe [10] described the possibility of dental abnormalities of the morphodifferentiation type, both numeric and of form, but did not specify the incidence. They hypothesized that a deficient number of teeth, including diffused or generalized hypodontia, is the most frequent anomaly and pointed out that this should not be confused with impacted teeth. The delay in tooth eruption has been calculated to be from one to three years for teeth that normally erupt in the first decade of life and from one to ten years for teeth erupting in the second decade [11, 12].

The case reported here had 69.2% of all reported anomalies of dwarfism. The root fusion in the case, although noted as a “rare situation,” reveals an aspect that, in the authors' opinion, is yet to be included in the various descriptions: tooth fusion between two or more crowns has been reported, whereas in the present case fusion was limited to the roots, which had independent and autonomous crowns. This feature makes the case unique, and it is also of interest for the type of prosthetic rehabilitation performed.

From the patient's medical history, her pituitary dwarfism was not associated with any other particular disorder. Her stature was very short (about 1.20 cm); she had not received GH replacement therapy nor thyroid, gonadal, or corticoid replacements.

Only 15.3% of anomalies relating to dwarfism, described in the literature, were missing from the case. In particular, there was no displacement of the first molars from the shaft to the ascending branch of the mandible, and both median maxillary central incisors were present.

The interest of this case resides both in the irregular dental situation and in the success of the therapeutic plan. Prosthetic rehabilitation in the aesthetic zone is one of the most challenging tasks in modern dentistry, especially in patients with serious syndromes or hormonal dysfunction. In recent years, aesthetic dentistry has been facilitated by the advent of materials and resins with improved mechanical performance that combine aesthetic and functional characteristics. In this context, the metal most used is zirconium. This is a transition metal of shiny white color, with excellent physical properties including good resistance to corrosion, which is lighter than steel and whose hardness is comparable to copper [13]. These characteristics are appropriate for the field of dentistry in which the use of a substructure to support fixed prosthetic rehabilitation is increasingly widespread. The result is a conservative therapeutic approach that does not intervene in the dental anomaly (asymptomatic); rather it is used as the support for a fully fixed prosthetic device. Surgery was thus avoided, in the form of root canal treatment (and retreatment) which would have compromised the stability and aesthetics of the prosthesis [14, 15].

With this conservative strategy, however, frequent follow-ups are essential: the fixity of the zirconium to the underlying teeth and the lack of periapical lesions of the fused roots must be continually monitored.

The report shows that, in patients with specific functional and aesthetic problems, including those with rare dental malformations, a good prosthetic rehabilitation can be achieved without resorting to surgery, thus avoiding any related complications, as well as the inevitable trauma to the patient.

## Figures and Tables

**Table 1:** Incidence of dental anomalies in pituitary dwarfism resulting from a search of PubMed for “oral cavity hypopituitarism” (ordered from most to least frequent abnormalities).

Oral abnormalities in pituitary dwarfism	Reported percentage incidence	Presence/absence in the case reported
Periodontal disease	98%	Present
Orthodontic malocclusion	95%	Present
Small size of the maxilla and mandible with overcrowding of teeth	95%	Present
Retention of permanent teeth in maxillary and mandibular shafts	75%	Present
Marked delay in eruption of permanent teeth	72%	Present
Delayed shedding of deciduous teeth	69%	Undefined
Solitary median maxillary central incisor	59%	Absent
No resorption of roots of deciduous teeth at the usual time	55%	Undefined
Development of apical parts of roots of retained permanent teeth and their growth toward the lower mandibular edge	55%	Present
Tilting of some of the retained teeth	35%	Present
Displacement of first molars from the shaft to the ascending branch of the mandible	10%	Absent
Complete absence of buds of wisdom teeth, even in patients in the fourth decade of life	10%	Present
Radicular fusion with crowns completely separate and independent	0%	Present
